# Biliary peritonitis due to liver cyst rupture in autosomal dominant polycystic kidney disease

**DOI:** 10.1186/s12876-021-01845-y

**Published:** 2021-06-24

**Authors:** Hiroshi Matsuo, Kan Katayama, Aoi Hayasaki, Yusuke Iizawa, Mayumi Endo, Tomohiro Murata, Shugo Mizuno, Kaoru Dohi

**Affiliations:** 1 Kidney Center, Suzuka Kaisei Hospital, Suzuka, Japan; 2 Department of Cardiology and Nephrology, Mie University Graduate School of Medicine, 2-174 Edobashi, Tsu, Mie 514-8507 Japan; 3 Department of Hepatobiliary Pancreatic and Transplant Surgery, Mie University Graduate School of Medicine, Tsu, Japan

**Keywords:** Autosomal dominant polycystic kidney disease, Biliary peritonitis, Liver cyst rupture

## Abstract

**Background:**

Autosomal dominant polycystic kidney disease (ADPKD) is the most frequent genetic kidney disease and polycystic liver disease is its major extrarenal manifestation, however biliary peritonitis due to a liver cyst rupture is extremely rare.

**Case presentation:**

The patient was a 71-year-old Japanese woman who was diagnosed with ADPKD 3 years previously and developed right abdominal pain suddenly 1 month previously. As abdominal computed tomography (CT) showed a ruptured liver cyst in the right lobe, she was admitted to our hospital. Her symptoms improved with conservative management and she was discharged from the hospital after 1 week. Although she was asymptomatic for a while, she noticed abdominal distension and general malaise at 1 month after hospital discharge. Since abdominal CT showed massive ascites, she was admitted to our hospital again. A physical examination revealed abdominal distention without tenderness. Her serum creatinine, alkaline phosphatase, γ-glutamyl transpeptidase, total bilirubin, and CA19-9 were elevated. Abdominal paracentesis revealed amber transparent ascites and the bilirubin and CA19-9 concentrations were high. She was diagnosed with biliary peritonitis due to a ruptured liver cyst. Hemodialysis treatment was initiated with drainage of the ascites. The outflow of the ascites was no tendency to decrease and drip infusion cholangiography (DIC)-CT revealed a communication between the ruptured cyst and an intrahepatic bile duct. On day 31, she was transferred to a university hospital and abdominal surgery was performed. After removing the necrotic roof of the ruptured cyst on the right liver lobe, the orifice of the bile leakage was sutured. Cholecystectomy was performed and cholangiography showed no stones in the common bile duct. Abdominal CT one month after the operation showed no recurrence of ascites and she was discharged on day 49. Hemodialysis treatment was discontinued immediately after discharge because urine volume increased and her creatinine level decreased. There has been no recurrence of ascites since then.

**Conclusions:**

While rare, biliary peritonitis can occur in association with the rupture of a liver cyst in ADPKD patients due to communication between the cyst and the intrahepatic bile duct, and DIC-CT should be recommended when biliary cyst rupture is suspected.

## Background

Autosomal dominant polycystic kidney disease (ADPKD) is the most frequent genetic kidney disease; the prevalence is 1 in 500–1000 [1]. There are two major causative genes: PKD1 and PKD2. The onset of end-stage kidney disease due to multiple cysts in both kidneys is much slower in patients with PKD2 mutation than in those with PKD1 mutation [2].

Extrarenal manifestations, include polycystic liver disease (PLD), intracranial aneurysm, mitral valve prolapse, and colon diverticulosis [3]. While PLD due to ADPKD is basically asymptomatic, acute symptomatic complications of single liver cysts can occur, including cyst infection, hemorrhage, torsion, or rupture [4]. We experienced a rare case of biliary peritonitis due to the rupture of a liver cyst in a patient with ADPKD, which was successfully treated with abdominal surgery.

## Case presentation

**Fig. 1:**
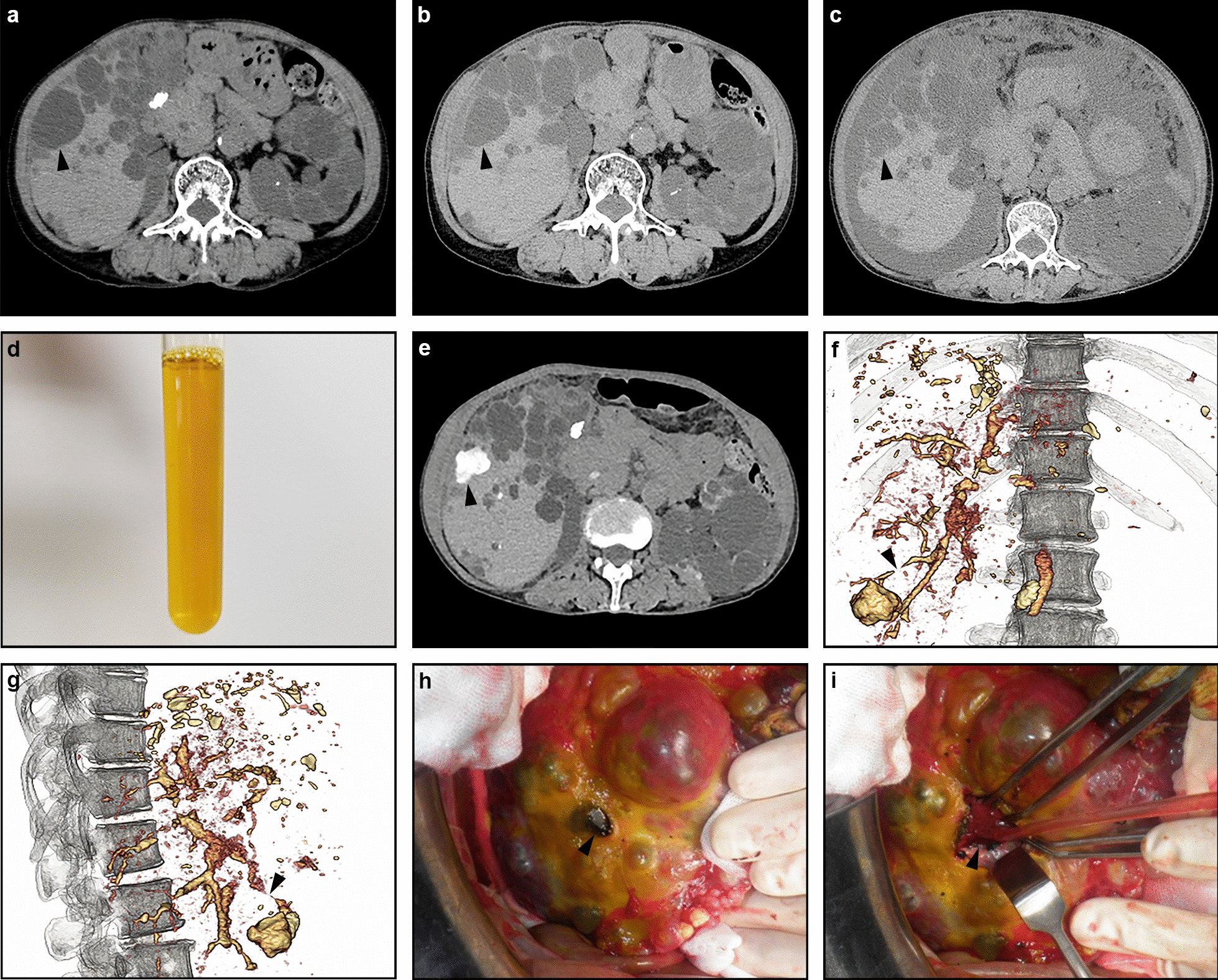
**a** Abdominal computed tomography (CT) 7 months previously showed gallbladder stones and multiple cysts in the liver and the left kidney. **b** Abdominal CT at the time of the first admission showed a ruptured liver cyst in the right lobe (arrowhead) while it was not observed seven months previously (arrowhead). **c** Abdominal CT on the second admission showed that the ruptured cyst (arrowhead) was reduced with massive ascites. **d** Abdominal paracentesis revealed amber transparent ascites. **e** Drip infusion cholangiography (DIC)-CT revealed that the ruptured cyst in the right posterior segment S6 of the liver was enhanced with contrast medium (arrowhead). **f**,** g** Three-dimensional (3D) views of DIC-CT showed communication between the ruptured cyst and the intrahepatic bile duct (arrowheads, **f**; front view, **g**; lateral view). **h** The surface of the liver became yellowish due to bile leakage and a ruptured necrotic cyst (arrowhead) was observed on the right liver lobe. **i** After removing the necrotic roof, the orifice (arrowhead) that was the source of the bile leakage was identified

The patient was a 71-year-old Japanese woman who was diagnosed with chronic kidney disease due to ADPKD 3 years previously. She received surgical clipping for an unruptured aneurysm of the right middle cerebral artery 4 years earlier. She did not drink alcohol and was a non-smoker. She was taking amlodipine for hypertension, topiroxostat for hyperuricemia, and ferric citrate hydrate for hyperphosphatemia. Continuous erythropoietin receptor activator was administered subcutaneously for renal anemia once a month. She was followed up in an outpatient clinic and suddenly developed right abdominal pain one month previously. As abdominal computed tomography (CT) showed a ruptured liver cyst in the right lobe, which had not been observed seven months previously (Fig. 1a, b), she was admitted to our hospital. While her white blood cell count was within the normal range at 5900/µL, C-reactive protein was a little high at 0.4 mg/dL. She was treated with bed rest and intravenous cefmetazole administration (1 g/day) on suspicion of cyst infection. Oral acetaminophen 500 mg was occasionally used when needed, and her symptoms improved with conservative management and she was discharged from the hospital after one week. Although she was asymptomatic for a while, she noticed bilateral leg edema, abdominal distension, and general malaise at one month after hospital discharge. Since abdominal CT showed massive ascites (Fig. 1c), she was admitted to our hospital again.

On admission, her height was 166 cm, and her body weight was 62 kg. A physical examination revealed the following: body temperature, 37.2 °C; blood pressure, 114/73 mmHg; pulse, 94 beats/min; and oxygen rate, 97% on room air. Abdominal distention was observed without tenderness, and pitting edema was observed in the bilateral legs. The laboratory data are shown in Table 1. Her serum creatinine, alkaline phosphatase, γ-glutamyl transpeptidase, total bilirubin, and CA19-9 were 5.2 mg/dL, 1992 U/L, 355 U/L, 3.1 mg/dL, and 5006 U/mL, respectively.

**Table 1:** Laboratory data on admission

Parameter	Patient value	Reference
*Urinalysis*		
Protein	(−)	(−)
Occult blood	1 +	<(+−)
*Hematology*		
White blood cells (/µL)	4600	4000–9000
Red blood cells (× 10^4^/µL)	332	380–480
Hemoglobin (g/dL)	10.5	12.0–16.0
Hematocrit (%)	32.1	34.0–42.0
Platelets (× 10^4^/µL)	12.6	12.0–40.0
*Coagulation*		
Activated partial thromboplastin time (s)	29	21–36
Prothrombin time international normalized ratio	1.06	0.88–1.08
Fibrinogen (mg/dL)	568	200–400
*Blood chemistry*		
Total protein (g/dL)	6.7	6.5–8.5
Albumin (g/dL)	2.8	4.1–5.3
Blood urea nitrogen (mg/dL)	47.1	9.0–20.0
Creatinine (mg/dL)	5.21	0.50–1.10
Aspartate aminotransferase (U/L)	27	5–40
Alanine aminotransferase (U/L)	12	4–44
Lactic dehydrogenation enzyme (U/L)	182	110–225
Alkaline phosphatase (U/L)	1992	120–340
γ-Glutamyl transpeptidase (U/L)	355	0–60
Total bilirubin (mg/dL)	3.1	0.2–1.3
Direct bilirubin (mg/dL)	2.6	0.0-0.5
Uric acid (mg/dL)	6	2.5-7.0
Natrium (mmol/L)	135	136–148
Potassium (mmol/L)	4	3.5-5.0
Chlorine (mmol/L)	99	98–110
Calcium (mg/dL)	8.2	8.5–10.5
Inorganic phosphate (mg/dL)	4.3	2.5–4.5
C-reactive protein (mg/dL)	4.97	< 0.30
Total cholesterol (mg/dL)	129	150–219
Triglyceride (mg/dL)	51	50–150
*Serological tests*		
HBs antigen	(−)	(−)
Anti-HCV antibody	(−)	(−)
CA19-9 (U/mL)	5006	0–37
*Ascites*		
Total protein (g/dL)	2.9	
Albumin (g/dL)	1.3	
Total bilirubin (mg/dL)	13.3	
Adenosine deaminase (U/L)	7.9	5–20
CA19-9 (U/mL)	244,700	

Abdominal paracentesis revealed amber transparent ascites (Fig. 1d) and the bilirubin and CA19-9 concentrations were high at 13.3 mg/dL and 244,700 U/mL, respectively. Ascites culturing and cytology were negative. She was diagnosed with biliary peritonitis due to a ruptured liver cyst. Hemodialysis was initiated due to the exacerbation of chronic kidney disease. As conservative treatment did not improve her symptom, drainage of the ascites was initiated from day 11. The outflow of the ascites was 1000 mL per day and there was no tendency to decrease. Drip infusion cholangiography (DIC)-CT revealed that the ruptured cyst in the right posterior segment S6 of the liver was enhanced with contrast medium (Fig. 1e). Three-dimensional views of DIC-CT showed communication between the ruptured cyst and an intrahepatic bile duct (Fig. 1f, g). Because of her markedly high CA19-9 levels in serum and ascites, upper endoscopy and repeated ascites cytology were required to rule out cancer. On day 24, oral cefditoren pivoxil (100 mg/day) was initiated because her C-reactive protein was still high at 7.4 mg/dL. On day 31, she was transferred to a university hospital, and the ascites culture on the same day was found to be positive for *Enterococcus faecalis*. Abdominal surgery was performed. The surface of the liver became yellowish due to bile leakage and a ruptured necrotic cyst was observed on the right liver lobe (Fig. 1 h). After removing the necrotic roof, the orifice that was the source of the bile leakage was identified and sutured (Fig. 1i). As gallbladder stones had been pointed out, cholecystectomy was performed at the same time. Cholangiography showed no stones in the common bile duct. Intravenous flomoxef sodium (0.5 g/day) was initiated for 3 consecutive days, followed by intravenous sulbactam/ampicillin (1.5 g/day) for 12 days. Abdominal CT one month after the operation showed no recurrence of ascites and she was discharged on day 49. Hemodialysis treatment was discontinued immediately after discharge because her urine volume had increased and her creatinine level had decreased to 3.8 mg/dL. There has been no recurrence of ascites since then.

## Discussion and conclusions

We experienced a rare case of biliary peritonitis due to a ruptured liver cyst in an ADPKD patient. While the first episode of liver cyst rupture improved with conservative management, the second episode caused massive ascites. Abdominal paracentesis was useful for confirming the diagnosis of biliary peritonitis, which was successfully treated with abdominal surgery.

Liver cysts are asymptomatic in most settings and liver cyst rupture is rare [5]. Marion et al. reviewed 11 cases of hemorrhagic liver cyst rupture, of which 4 cases involved patients with ADPKD [6]. Three of the four cases had a hemodynamic impact and two of the four cases were fatal [7–10]. There was also a case of fatal liver cyst rupture in a patient with ADPKD due to trauma [11]. Another report showed that acute abdomen and ascites due to liver cyst rupture led to the diagnosis of ADPKD [12]. However, to the best of our knowledge, there have been no reports on biliary peritonitis due to spontaneous liver cyst rupture in a patient with ADPKD.

While ruptures of the gallbladder, common bile duct [13], extrahepatic bile duct [14], and intrahepatic bile duct [15, 16] are considered to be causes of spontaneous biliary peritonitis, the development of peritonitis in association with the rupture of a liver cyst communicating with a biliary tract is rare. Three-dimensional DIC-CT showed dilatations of the distal intrahepatic bile ducts near the liver surface, suggesting partially increased internal pressure of the bile duct with biliary stasis. There have been reported cases involving a liver cyst with biliary communication and both cases were treated by laparoscopic deroofing before rupture [17, 18]. The cystobiliary communication was found by percutaneous transhepatic drainage of the cyst before the operation in one report [17], while it was recognized during laparoscopic deroofing in the other report [18]. The difficulty in finding cystobiliary communication might be attributed to the high intracystic pressure [18]. While the first ascites culture was negative in the present case, the existence of bile in the abdominal cavity increased the risk of secondary bacterial infection [19]. Indeed, the second ascites culture before operation was positive for *Enterococcus faecalis*. Abdominal surgery instead of laparoscopy, or non-operative approaches, such as percutaneous transhepatic biliary drainage, was chosen in the present case for several reasons. First, massive leakage of the bile was suspected because the amount of biliary ascites exceeded 1000 mL per day. Second, the ascites culture was positive for *E. faecalis* before operation. Third, the ruptured cyst was a small cyst located among multiple cysts, so there was a possibility that the orifice would not be visible under laparoscopy. Abdominal surgery to close the orifice that was the source of the bile leakage in the ruptured cyst achieved a favorable outcome without severe bacterial infection in the present case. We do not think the present case was indicated for liver transplantation, as closure of the orifice of the bile leakage was successful; however, liver transplantation may be a viable option in cases of repetitive biliary peritonitis due to many hepatobiliary communications after controlling the infection and taking into account the patient’s age and other comorbidities.

In conclusion, while rare, biliary peritonitis can occur in association with the rupture of a liver cyst in ADPKD patients due to communication between the cyst and the intrahepatic bile duct, and DIC-CT should be recommended when biliary cyst rupture is suspected.

## Data Availability

The datasets used and/or analyzed during the current study are available from the corresponding author on reasonable request.

## Abbreviations

ADPKD: Autosomal dominant polycystic kidney disease
CT: Computed tomography
DIC: Drip infusion cholecystocholangiography
PLD: Polycystic liver disease
